# Phosphatidylserine exposing-platelets and microparticles promote procoagulant activity in colon cancer patients

**DOI:** 10.1186/s13046-016-0328-9

**Published:** 2016-03-25

**Authors:** Liangliang Zhao, Yayan Bi, Junjie Kou, Jialan Shi, Daxun Piao

**Affiliations:** Department of Colorectal Surgery, the First Affiliated Hospital of Harbin Medical University, 23 Youzheng Street, Nangang District, Harbin, Heilongjiang Province, 150001 People’s Republic of China; Department of Medicine, the First Affiliated Hospital of Harbin Medical University, 23 Youzheng Street, Nangang District, Harbin, Heilongjiang Province, 150001 People’s Republic of China; Department of Cardiology, the Second Affiliated Hospital of Harbin Medical University, 246 Xuefu Road, Nangang District, Harbin, Heilongjiang Province, 150086 People’s Republic of China; Department of Surgery, Brigham and Women’s Hospital, VA Boston Healthcare System, Harvard Medical School, Boston, 02132 USA; Department of Hematology, the First Affiliated Hospital of Harbin Medical University, 23 Youzheng Street, Nangang District, Harbin, Heilongjiang Province, 150001 People’s Republic of China

**Keywords:** Colon cancer, Phosphatidylserine, Platelets and microparticles, Procoagulant activity

## Abstract

**Background:**

Colon cancer is invariably accompanied by altered coagulation activity; however, the precise role of phosphatidylserine (PS) in the hypercoagulable state of colon cancer patients remains unclear. We explored the exposure of PS on platelets and microparticles (MPs), and evaluate its role in procoagulant activity in colon cancer patients.

**Methods:**

PS-positive platelets and MPs, mainly from platelets and endothelial cells, were detected by flow cytometry and confocal microscopy, and their procoagulant activity was assessed with purified coagulation complex assays, clotting time, and fibrin turbidity.

**Results:**

Plasma levels of PS-positive platelets increased gradually from stage I to IV and were higher in all stages of the patients than in the healthy control, while PS-positive platelet-derived MPs only increased significantly in stage III/IV patients. Meanwhile, PS-positive MPs and endothelial-derived MPs in stage II/III/IV patients were markedly higher than ones in controls but no difference with stage I. Tissue factor positive MPs were higher in all 4 stages of colon cancer patients than in the healthy control. Platelets and MPs from the patients demonstrated significantly enhanced intrinsic/extrinsic FXa and thrombin generation, greatly shortened coagulation time, and increased fibrin formation. Combined treatment with PS antagonist lactadherin, strongly prolonged the coagulation time and reduced fibrin formation by inhibiting factor tenase and prothrombinase complex activity. In contrast, pretreatment with anti tissue factor antibody played a lesser role in suppression of procoagulant activity.

**Conclusion:**

Our results suggest that PS-positive platelets and MPs contribute to hypercoagulability and represent a potential therapeutic target to prevent coagulation in patients with colon cancer.

## Background

Colorectal cancer is the most common gastrointestinal cancer and the second cause of cancer death worldwide, with the incidence of colon cancer increasing in most countries over the past 20 years [1, 2]. Thrombotic disorders including venous thromboembolism, a life-threatening complication, were frequently observed in patients with colon cancer [3, 4] . Previous studies have demonstrated the development of a hypercoagulable state in colon cancer patients with elevated markers of coagulation, including thrombin-antithrombin complex, prothrombin fragment 1 + 2, soluble fibrin, and total fibrin(ogen) degradation products fibrinogen [5, 6]. However, relatively little is known about the mechanism of this increased procoagulant activity (PCA) in those patients. Increased activity of the coagulation cascade and decreased activity of coagulation inhibitors (e.g., tissue factor pathway inhibitor) at the colon cancer site were also demonstrated [7, 8]. Nevertheless, circulating cells or other factors that are associated with this hypercoagulable state in colon cancer have not been well determined.

Phosphatidylserine (PS), on the surface of activated platelets, confers a procoagulant surface necessary for hemostasis by providing binding sites for both intrinsic and extrinsic FXa and the assembly of prothrombinase complexes to generate thrombin, resulting in fibrin formation [9, 10]. Others and our previous studies have shown that elevated PS exposure on circulating platelets plays a critical role in the thrombotic risk associated disorders, including chronic uremia, nephrotic syndrome, and polycythemia vera [11–13]. Platelet hyperactivation has been observed in colon cancer and is indicated to be involved in the cancer progression [14–16]. However, relatively little is known about to what extent PS exposed on platelets, or whether PS^+^ platelets contribute to over-exuberant coagulation in different stage of colon cancer patients.

PS express at the surface of circulating microparticles (MPs), which are small vesicular structures (0.1-1 μm diameter) produced and released by exocytic blebbing of the activated cell plasma membrane from a variety of cell types such as platelets and endothelial cells [17, 18]. Although there have been study using annexin V as a probe to detect PS^+^ MPs in colon cancer patients [19], inhibition assays were not performed to clarify whether PS^+^ MPs are responsible for MP-associated PCA. Studies also implicated that tissue factor (TF) may be the pivotal source for the generation of MP and increased TF activity of MP has been inducted in colon cancer patients [19, 20]. However, exposed TF is generally quiescent unless it resides in a membrane containing PS [21]. The relative contributions of TF and PS to coagulation activation in colon cancer patients remain to be established.

Our previous study have demonstrated that lactadherin was a more sensitive probe than annexin V for the detection of PS exposure on circulating cells or MPs, and it could be an anticoagulant to abrogate activation of the coagulation cascade both in vitro and vivo [22–24]. Therefore, we used lactadherin to quantify and further analyzed PS^+^ platelets and MPs in different stage of colon cancer patients. We defined the intrinsic and extrinsic FXa, and thrombin generation, coagulation time, and fibrin formation of the PS^+^ platelets and MPs in the study subjects. Lactadherin and anti-TF antibody was used to inhibit the PCA of the platelets and MPs. The current study enables us to better understand the mechanism of hypercoagulable state in colon cancer patients.

## Methods

### Patients

From October 2014 to November 2015, 112 colon cancer patients, admitted to the First Hospital of Harbin Medical University, were enrolled in the study. Patients were all diagnosed with colon cancer by pathological examinations through colonoscopy. Then blood samples of diagnosed patients were collected before the operation and chemotherapy for experiments. Those who needed surgical operation were further diagnosed by pathological examinations during surgical operation to clarify whether those two results were consistent. Patients who had previously received anticoagulation or antiplatelet therapy such as aspirin, or had a history of coagulation or hemorrhage complications within three months were excluded from the study. Other exclusion criteria included diabetes, hypertension, malignant or systemic disease, pregnancy, active or chronic infection, blood transfusion within the past six months. The patient group consisted of 55 males and 57 females, with ages ranging from 36 to 83 years. The disease stage for the patients was confirmed according to cancer staging criteria of the 7th edition staging American Joint Committee on Cancer (AJCC) [25]. The control group was composed of 33 healthy blood donor volunteers (17 males and 16 females). They were chosen from routine health examinations based on the following selection criteria: all physical indicators were in the normal range and the volunteers were free of cancer, hepatitis, or infection, among others. Characteristics of all the study subjects are shown in Table 1. The protocols and procedures were approved by the Harbin Medical University research ethics committee and were based on the guiding policy, and informed consent was obtained from all participants.

**Table 1:** Baseline characteristics of patients with colon cancer and healthy subjects at inclusion

Characteristics	Healthy subjects (*n* = 33)	Stage I (*n* = 16)	Stage II (*n* = 47)	Stage III (*n* = 28)	Stage IV (*n* = 21)
Gender (Male, %)	17 (51.52 %)	6 (37.50 %)	29 (61.70 %)	12 (42.86 %)	8 (38.10 %)
Age (years)	60.72 ± 10.26	61.38 ± 11.71	63.51 ± 10.50	64.38 ± 9.99	58.86 ± 12.00
Albumin (g/l)	38.91 ± 4.87	33.96 ± 6.71	36.22 ± 6.22	36.99 ± 6.25	36.97 ± 4.62
Total cholesterol (mM)	3.96 ± 0.62	3.93 ± 1.14	3.85 ± 1.24	3.50 ± 0.89	3.48 ± 1.17
Triglycerides (mM)	0.94 ± 0.46	1.26 ± 0.35	1.37 ± 0.80	1.31 ± 0.66	1.67 ± 0.61
CEA (ng/ml)	ND	2.13 (1.08-2.30)	4.73 (3.23-18.88)^b^	3.16 (2.55-7.18)^b^	10.54 (8.91-13.64)^bcd^
CA199 (U/ml)	ND	9.45 (5.38-10.19)	15.76 (10.73-34.51)^b^	19.43 (10.67-46.56)^b^	64.07 (2.56-779.92)^bcd^
Platelet count (10^9^/L)	248.35 ± 50.32	281.40 ± 94.95	259.62 ± 91.42	273.79 ± 87.21	309.97 ± 77.95
Erythrocyte count (10^12^/L)	4.35 ± 0.42	4.16 ± 0.71	4.43 ± 0.83	4.50 ± 0.59	4.23 ± 0.84
Hemoglobin (g/L)	126.49 ± 20.37	117.51 ± 32.60	123.81 ± 30.15	124.73 ± 27.60	120.23 ± 29.76
PT (s)	11.70 ± 0.72	10.83 ± 0.70	11.86 ± 0.92	11.26 ± 0.87	11.06 ± 0.81
APTT (s)	31.41 ± 2.85	27.14 ± 3.41^a^	27.84 ± 4.01^a^	25.39 ± 2.85^abc^	24.81 ± 2.56^abc^
D-dimer (mg/L)	0.13 (0.06-0.18)	0.31 (0.19-1.07)^a^	0.38 (0.22-1.78)^a^	0.72 (0.32-1.16)^abc^	0.73 (0.29-1.12)^abc^
Fibrinogen (g/L)	3.47 ± 1.03	3.59 ± 1.66	3.89 ± 1.19	3.42 ± 0.72	3.81 ± 0.58
Current smoking, n (%)	5 (15.15 %)	2 (12.50 %)	9 (19.15 %)	6 (21.43 %)	5 (23.81 %)
Thrombotic events, n (%)	2 (6.06 %)	3 (18.75 %)^a^	10 (21.28 %)^a^	9 (32.14 %)^a^	6 (28.57 %)^a^

Data are expressed by mean ± standard deviation [SD], percentage or median (interquartile range [IQR]). *PT* prothrombin time, *APTT* activated partial thromboplastin time. *ND* not determined. ^a^ *P* < 0.05 versus healthy controls, ^b^ *P* < 0.05 versus stage I, ^c^ *P* < 0.05 versus stage II. ^d^ *P* < 0.01 versus stage III

### Materials

Calibrated polystyrene latex beads (1.0 μm) were from Sigma (UK). Trucount Tube (Cat. No. 340334), purified CD31 (clone L133.1), CD41a (clone HIP8), CD142 (clone HFT-1), and mouse IgG1/IgG2a (clone X40/X39) were from Becton Dickinson Biosciences (San Jose, CA, USA). All monoclonal antibodies were labelled in our laboratory with Alexa Fluro 647 or Alexa Fluro 488. Polyclonal antibody against human Tissue Factor (product No. 4502) was from American Diagnostica Inc. (Stamford, CT, USA). Alexa Fluro 647-conjugated lactadherin were prepared in our laboratory. Human factors Va, VIIa, VIII, IXa, X, Xa, prothrombin and thrombin were all from Haematologic Technologies (Burlington, VT, USA). Mouse anti-fibrin II chain (clone NYBT2G1) was from Accurate Chemical & Scientific (Westbury, NY, USA). Isotype control antibody was from Dako (Carpinteria, CA, USA). Tyrode’s buffer containing 1 mM Hepes was constituted in our laboratory and was filtered through a 0.22 μm syringe filter from Millipore (UK). Chromogenic substrates S-2765 and S-2238 were from DiaPharma Group (West Chester, OH, USA).

### Protein purification and labeling

Lactadherin was purified from bovine milk and labeled with Alexa Fluor 647 or Alexa Fluor 488 as described previously. The ratio of fluorescein to lactadherin was 1.2/1 or 1.1/1 [24] .

### Blood collection, preparation of platelets and MPs

Blood was drawn before therapy with a 21-gauge needle and was collected into a 5-mL tube containing 3.2 % citrate (BD, Plymouth, UK). Platelet-rich plasma (PRP) was prepared within 30 min (min) of blood collection by centrifugation for 13 min, 200 g at room temperature, and was analyzed immediately after isolation. Platelet-free plasma (PFP) was prepared as described [13, 26]. Briefly, samples were centrifuged for 20 min at 1,500 g, and plasma was harvested and re-centrifuged for 2 min at 13,000 g to remove all residual platelets. PFP were snap-frozen in liquid nitrogen, and then stored at −80 °C until use. In order to isolate the MPs, 250 μl of PFP was thawed on ice for 60 min and then centrifuged at 20,000 g for 45 min at 20 °C [13, 27]. Subsequently, 225 μl of supernatant (i.e. MP-free plasma) was aspirated and the remaining 25 μL MPs pellet was washed once by centrifugation and resuspended in 75 μl of Tyrode’s buffer (MP enriched suspension).

### Flow cytometric analysis of PS exposure on platelets

The exposure of PS on platelets was measured using lactadherin binding by flow cytometry. Platelets were adjusted to (0.5-1) × 10^6^/ml in a final volume of 200 μl with Tyrode’s buffer; five μl of Alexa 488-conjugated lactadherin was added to the cell suspension and incubated for 10 min at room temperature in the dark. Five thousand events per sample were acquired and analyzed with BD FACS Diva Software.

### Flow cytometric analysis of PS exposure on MPs

MPs were identified as described previously [13]. Platelet, endothelial and tissue factor (TF)-derived MPs were defined as smaller than 1 μm and lactadherin^+^ CD41a^+^, lactadherin^+^ CD31^+^CD41a^−^ and lactadherin^+^ CD142^+^, respectively. The number of each MP type per μl was calculated by Trucount Tube (with a precise number of fluorescent beads 48678 to determine the number of MPs in a sample) after accumulation of 5,000 gated events.

### FXa and prothrombinase formation and inhibition assays

The formation of intrinsic and extrinsic FXa and prothrombinase in the presence of platelets and MPs was performed as previously described [13]. For the intrinsic FXase formation assays, 1 × 10^4^ platelets or 10 μl of MP suspension (prepared as described above) were incubated with 1 nM FIXa, 130 nM FX, 0.2 nM thrombin, and 5 nM FVIII in FXa buffer (1 ml 10 × TBS, 200 μl 10 % BSA, 8.8 ml ddH_2_O) at 25 °C for 5 min followed by addition of EDTA (7 mM, final concentration) to stop the reaction. Immediately after the addition of 10 μl chromogenic substrate S-2765 (0.8 mM), production of FXa was determined by measurement of absorbance at 405 nm on a Universal Microplate Spectrophotometer (PowerWave XS, Bio-Tek, Winooski, VT, USA) in kinetic mode. The measurement lasted 15 min, and the read interval was 11 s. Measurement of extrinsic FXa formation was analogous to that for intrinsic FXa except that cells or MPs were incubation with 130 nM FX, 1nM FVIIa and 5 mM Ca^2+^. Each test was performed in triplicate. Results were measured against the rate of substrate cleavage of a standard dilution of FXa. The means of generated FXa for all time points were analyzed.

In the prothrombinase formation assay, cells or MPs were incubated with 1 nM FVa, 0.05 nM FXa in the presence of 1 μM prothrombin and 5 mM Ca^2+^ in prothrombinase buffer (1 × TBS with 0.05 % BSA) at 25 °C for 5 min. After addition of EDTA and chromogenic substrate S-2238 (0.8 mM), thrombin production was assessed as described in the FXase formation assay. Results were evaluated against the rate of substrate cleavage from a dilution curve of thrombin. To test the inhibition of coagulation complexes by lactadherin or anti-TF antibody, the platelets or MPs-containing suspension was preincubated with lactadherin (128 nM) or anti-TF (40 μg/ml) for 10 min at 25 °C in Tyrode’s buffer. The mixture was then incubated with the specified clotting factors according to the above protocols. The quantity of FXa or thrombin formation was assessed as previously described [13, 24].

### Coagulation time and fibrin formation and inhibition assay of platelets and MPs

PCA of platelets and MPs was evaluated by one-stage recalcification time assay in a KC4A-coagulometer (Amelung, Labcon, Heppenheim, Germany). One hundred μl of platelet (1 × 10^7^) or MPs suspension (10 μl of MPs-enriched suspension was resuspended in 90 μl Tyrode’s buffer) was incubated with 100 μl of MP-free human plasma at 37 °C. After 180 s, 100 μl of warmed 1.5 mM CaCl_2_ was added to start the reaction and the clotting time was recorded. All clotting assays were performed in triplicate. Based on the previous analysis, we selected the stage IV of the colon cancer patients to perform inhibition assays of platelets and MPs. Fifty μl of lactadherin (128 nM) or anti-TF (40 μg/ml) was added to 100 μl cells or MP suspension, and then incubated for 10 min at 37 °C. Clotting time was recorded after adding of 100 μl MP-free human plasma and 50 μl of warmed 1.5 mM CaCl_2_ as mentioned above.

Fibrin formation was quantified by turbidity as described [28]. Isolated platelets and MPs (as used in coagulation time assays) were added to re-calcified (10 mM, final) MDP (88 % MDP, final) in the circumstance of MDP isolated from healthy donors in the presence or absence of lactadherin (128 nM) or anti-TF (40 μg/ml). Fibrin formation was measured by turbidity at 405 nm in a SpectraMax 340PC plate reader. Each test was performed in triplicate.

### Confocal microscopy

To evaluate and image PS exposure, platelets (colon cancer stage IV) were incubated with Alexa 488-lactadherin (4 nM, final concentration) for 10 min at room temperature in the dark, then washed to remove unbound dye, and imaged immediately. To observe the contribution of MPs to fibrin formation in colon cancer stage IV, MPs-containing suspensions (25 μl MPs was washed twice and resuspended in 75 μl of Tyrode’s buffer) were incubated with plasma (platelet poor plasma centrifuged 106,000 g for 1 h at 4 °C) and 3 mM calcium. Fibrin networks were imaged using laser confocal microscopy in the presence of Alexa 647-conjugated anti-fibrin. Background signal was calculated using a similarly labeled isotype matched control antibody. Observation of the PS exposure on MPs labeled with Alexa Fluor 488-lactadherin and Alexa Fluro 647-annexin V was carried out by confocal microscopy as previously described [13]. Images were obtained in using LSM 510 SYSTEM (Carl Zeiss Jena GmbH, Jena, Germany).

### Statistical analysis

Numerical variables were tested for normal distribution with the Kolmogorov-Smirnov test. Normally distributed variables were summarized as mean (±SD) and compared using one-way-ANOVA followed by Bonferroni’s multiple comparison tests. Non-normally distributed variables were summarized as medians with interquartile ranges (IQR). Median values of clinical parameters and MP count of healthy subjects and colon cancer groups were compared using Kruskal-Wallis statistical test and further analysed using Dunn’s multiple comparison test. Frequencies were provided for all nominal values and differences were calculated using Chi-square test. Spearman’s rank correlation was run to determine the relationship between the colon cancer metastasis and PS^+^ platelets, MPs, PMPs, or EMPs levels, and between clotting time and those PS^+^ cells and MPs levels. All statistical analyses were carried out using Graph Pad Prism (version 5.0) or SPSS 16.0 statistical software package. *P* <0.05 was considered statistically significant.

## Results

### Subject characteristics

Clinical characteristics of healthy subjects (HS) and different stages of colon cancer patients are shown in Tables 1. One hundred and twelve patients with colon cancer, including 16 stage I, 47 stage II, 28 stage III and 21 stage IV, were investigated. For the 21 stage IV colon cancer patients, 12 patients have liver metastasis, 7 patients lung metastasis, 1 patient brain metastasis and the remaining one has bone metastasis. For each stage (I, II, III, and IV), thrombotic events happened more frequently in colon cancer patients than in healthy controls. In addition, the level of APTT in patients with colon cancer stage III/IV were significantly lower than those measured in stage I/II patients, with highest level in healthy individuals, while D-dimer had an inverse trend. Within subgroups of colon cancer patients, stage IV patients had significantly higher level of CEA and CA-199 than those in stage II/III, and lowest in stage I.

Compared with controls, different stages of colon cancer patients had no significant difference in gender, age, albumin, total cholesterol, triglycerides, platelet and erythrocyte counts, hemoglobin, PT, fibrinogen and current smoking. Moreover, we did not find positive relationships between MP levels and serum albumin, total cholesterol, triglycerides and hemoglobin. Nevertheless, we found a positive correlation between colon cancer metastasis and PS^+^ platelets (*r* = 0.51, *P* = 0.003), MPs (*r* = 0.27, *P* = 0.036), and PMPs levels (*r* = 0.39, *P* = 0.016) but not EMPs (*r* = 0.79, *P* = 0.182), or TF^+^ MPs levels (*r* = 0.54, *P* = 0.068).

### PS^+^ platelet levels

**Fig. 1:**
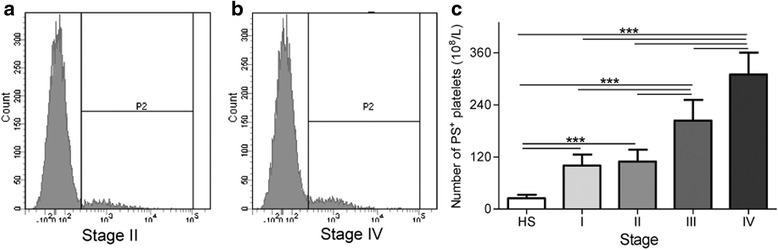
Phosphatidylserine (PS) exposure on platelets. Isolated platelets were incubated with Alexa Fluro 488-lactadherin separately in the dark for 10 min at room temperature before flow cytometric analysis. A representative set of histograms is shown to illustrate lactadherin-positive (PS^+^) platelets from (**a**) one stage II patient with colon cancer, and (**b**) one stage IV patient. **c** Comparison of PS^+^ platelets among healthy subjects (*n* = 33), and colon cancer patients in different stage (stage I: *n* = 16, II: *n* = 47, III: *n* = 28, or IV: *n* = 21). Data displayed are mean ± SD. ***P <0.001

We first measured the exposure of PS on the extracellular membrane of blood platelets in healthy subjects and colon cancer patients by flow cytometry. PS^+^ platelets in two colon cancer patients with stage II and IV respectively were shown in Fig. 1a and b. For each stage (I, II, III, and IV), the plasma levels of PS^+^ platelets [(100 ± 26) × 10^8^/L, (109 ± 27) × 10^8^/L, (204 ± 48) × 10^8^/L and (310 ± 51) × 10^8^/L, respectively] were significantly (*P* <0.001) higher in colon cancer patients than in HS [(25 ± 8) × 10^8^/L] (Fig. 1c). In addition, the plasma levels of PS^+^ platelets in patients with cancer stage IV were significantly (*P* <0.001) higher than those measured in stage I, II, and III patients. Patients with stage III have significantly (*P* <0.001) higher level of PS^+^ platelets than those in stage I and II, but no statistic difference (*P* >0.05) in PS^+^ platelets between stage I and II.

### Origin of MPs and their levels

**Fig. 2:**
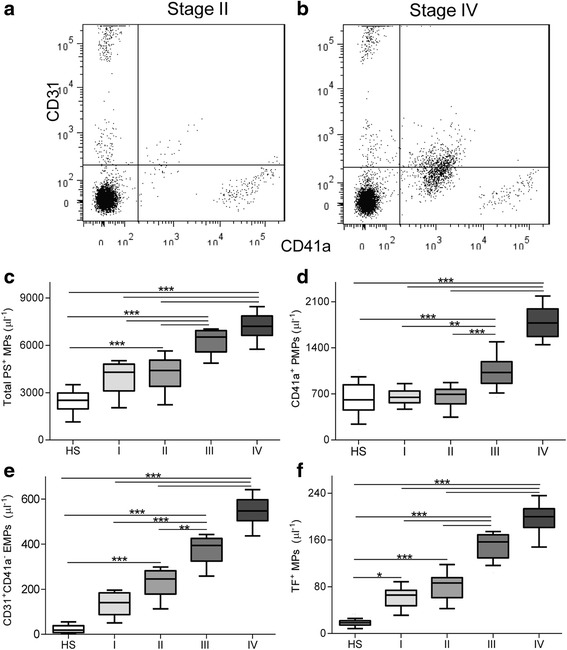
Number and origin of MPs by flow cytometry analyses in study subjects. A representative set of scattergrams in two samples from colon cancer patients is shown to illustrate MPs and subpopulation definitions. Lactadherin-positive MPs were examined for expression of other antigens by co-labeling with Alexa Fluor 488- and Alexa Fluor 647-labeled antibodies as is shown here for platelet-derived MPs (Alexa Fluro 488-CD41a^+^) and endothelia-derived MPs (Alexa Fluro 647-CD31^+^/Alexa Fluor 488-CD41a^−^) in (**a**) stage II or (**b**) IV of colon cancer patients. Comparison of **c** PS^+^ total MPs, **d** platelet-derived MPs (PMPs), **e** endothelial-derived MPs (EMPs), and **f** tissue factor-positive MPs (TF^+^ MPs) among healthy subjects (*n* = 33), stage I (*n* = 16), II (*n* = 47), III (*n* = 28), or IV (*n* = 21) colon cancer patients. Data are given as median (horizontal bar), 25th and 75th percentile (boxes), and 10th and 90th percentile (error bar). *P <0.05; **P <0.01; ***P <0.001

Total number of MPs and their phenotypic characterization were tested. A known count of larger beads (TruCount beads, Becton Dickinson) was used as an internal standard enabling us to calculate the absolute number of MPs per volume of specimen. For most study subjects, more than 90 % of events were PS positive as we previously described [13]. PMPs, EMPs and TF MPs are defined as platelet, endothelia and TF-derived MPs separately. MPs in colon cancer patients can be originating from platelets and endothelial cells (Fig. 2a and b).

Plasma levels of total PS^+^ MPs (lactadherin^+^ phenotype) were significantly (*P* <0.001) increased in patients with stage II, III, and IV [4405 (3402–5060) μl^−1^, 6525 (5579–6932) μl^−1^, and 7203 (6625–7858) μl^−1^, respectively] as compared with total PS^+^ MP levels measured in HS [2515 (1968–2977) μl^−1^] (Fig. 2c). Plasma levels of PS^+^ MPs observed in stage I patients [4298 (3115–4806) μl^−1^] were higher than those observed in HS, but with no significant difference. Although plasma levels of PS^+^ MPs in stage IV or III patients were significantly (*P* <0.001) higher than those in stage I or II, we did not found any significant difference between stage I and II (*P* >0.05), and between stage III and IV (*P* >0.05). PMPs (CD41a^+^ phenotype) had a similar trend. Plasma levels of PS^+^ PMPs in stage IV [1779 (1573–1992) μl^−1^] or III [1027 (861–1192) μl^−1^] patients were significantly (*P* <0.001) higher than those in HS [611 (457–839) μl^−1^], stage I [649 (567–744) μl^−1^], or II [696 (549–771) μl^−1^], but we did not found any significant difference between stage I and II (*P* >0.05), and between stage III and IV (*P* >0.05) (Fig. 2d).

Plasma levels of EMPs (CD31^+^CD41a^−^ phenotype) were significantly (*P* <0.01) increased in patients with stage II, III, and IV [245 (178–282) μl^−1^, 394 (325–425) μl^−1^ and 547 (504–597) μl^−1^, respectively] as compared with those measured in HS [18 (7–38) μl^−1^] (Fig. 2e). Plasma levels of EMPs observed in HS were lower than those observed in stage I patients [140 (87–184) μl^−1^], but with no significant difference (*P* >0.05). TF^+^ MPs (CD142^+^ phenotype) in patients with stage I, II, III and IV [66 (48–74) μl^−1^, 87 (61–96) μl^−1^, 157 (130–169) μl^−1^ and 200 (181–214) μl^−1^, respectively] had significantly (*P* <0.05) higher levels than those in HS [18 (14–22) μl^−1^] (Fig. 2f). Plasma levels of both EMPs and TF^+^ MPs in patients with stage III or IV were significantly (*P* <0.01) higher than those in patients with stage I or II, respectively. EMPs and TF^+^ MPs levels did not significantly differ between colon cancer patients stage I and II (*P* >0.05), and between patients stage III and IV (*P* >0.05).

### Formation and inhibition assays of procoagulant enzyme complexes of platelets and MPs

**Fig. 3:**
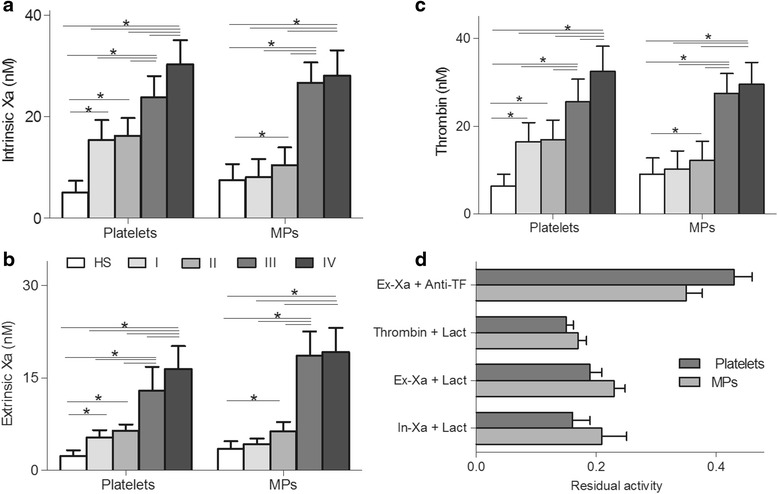
Procoagulant enzyme complexes formation and inhibition assays. **a** Intrinsic FXa, **b** extrinsic FXa, and **c** thrombin production of platelets and MPs from healthy subjects (*n* = 33), stage I (*n* = 16), II (*n* = 47), III (*n* = 28), or IV (*n* = 21) colon cancer patients were evaluated. Intrinsic FXa formation was measured in the presence of FIXa, FVIII and thrombin. Extrinsic FXa production was assessed in the presence of FVIIa. Thrombin generation was investigated in the presence of FXa and FVa. **d** The capacity of lactadherin (128 nM) to block procoagulant enzyme complexes on platelets and MPs from 21 patients stage IV was evaluated. lactadherin decreased activity of the procoagulant enzyme complexes by approximately 80 % in platelets. Data displayed are mean ± SD. *P <0.001. In-Xa: intrisic FXa; Ex-Xa: extrisic FXa; lact: lactadherin

Consistent with the above finding of increased PS exposure on platelets and MPs, these entities also demonstrated heterogeneous levels in intrinsic/extrinsic FXa and thrombin production (Fig. 3). Platelet intrinsic FXa, extrinsic FXa, and thrombin were significantly (*P* <0.001) higher in all stages of colon cancer patients than in HS (Fig. 3a-c). The production of the three-procoagulant enzyme complexes in platelets was significantly (*P* < 0.001) increased in patients with cancer stage IV compared with stage I, II, and III patients respectively. In addition, patients with stage III have obviously higher (*P* <0.001) level of platelet intrinsic and extrinsic FXa, and thrombin than those measured in stage I or II, but no statistic difference between stage I and II (*P* >0.05). MP FXa and thrombin were significantly (*P* <0.001) increased in patients with stage II, III, and IV as compared with that measured in HS (Fig. 3a-c). The three procoagulant enzyme complexes of MPs observed in stage I patients were higher (*P* <0.001) than those observed in HS, but with no significant difference (*P* >0.05). Although MP FXa and thrombin in stage IV or III patients were higher than those in stage I or II (*P* <0.001), we did not found any significant difference between stage I and II (*P* >0.05), and between stage III and IV (*P* >0.05).

To determine the necessity of exposed PS and TF on platelets and MPs to support procoagulant reactions, we performed the PCA inhibition assays using platelets and MPs from colon cancer patients with stage IV which had the most large amount of PS^+^ platelets or MPs. FXase and prothrombinase inhibition assays were performed with 128 nM lactadherin or 40 μg/ml anti-TF as we previously described [13]. The intrinsic FXa, extrinsic FXa and thrombin production for platelets were reduced by 84 %, 81 % and 85 %, and MPs by 79 %, 77 % and 83 %, when lactadherin were added (Fig. 3d). Meanwhile, the extrinsic FXa for platelets and MPs were reduced by 57 % and 64 % resperively when anti-TF antibody was included. Thus, lactadherin blocks procoagulant enzyme complex generation greater than anti-TF antibody.

### Coagulation and fibrin formation and inhibition assays of platelets and MPs

**Fig. 4:**
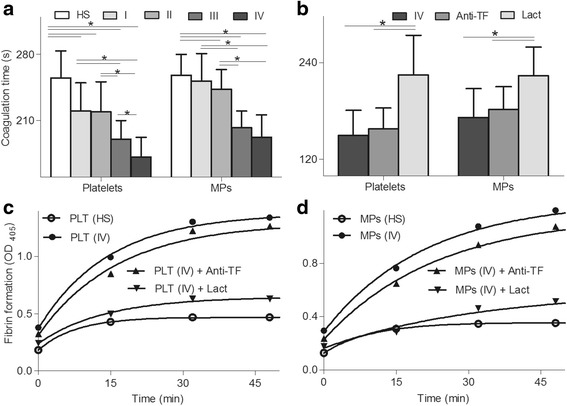
Coagulation and fibrin formation and inhibition assays. **a** Coagulation times of platelets and microparticles (MPs) from healthy subjects (*n* = 33), stage I (*n* = 16), II (*n* = 47), III (*n* = 28), or IV (*n* = 21) colon cancer patients were measured. **b** Coagulation time of platelets and MPs in 21 stage IV colon cancer patients was detected in the absence or presence of lactadherin (128 mM) or anti-TF (25.6 μg/ml). Fibrin production on **c** platelets or **d** MPs was detected in the presence of recalcified MDP with or without 128 nM lactadherin or 25.6 μg/ml anti-TF. Data displayed are mean ± SD. *P <0.05

The PCA of platelets and MPs was further assessed by recalcification-time assays in the study subjects (Fig. 4a). Platelets isolated from each stage of colon cancer patients had markedly shortened clotting time (*P* <0.05), compared with equal numbers of cells from control subjects. The coagulation time of MPs significantly (*P* <0.05) decreased in stage II, III and IV when compared to that in HS. Platelets and MPs coagulation time were significantly (*P* <0.05) short in patients with stage III or IV in comparison to stage I or II. Additionally, platelet coagulation time in stage IV were shorter (*P* <0.05) than those in stage III. However, no significant difference was found in stage III comparing to stage IV of MPs (*P* >0.05), and in stage I comparing to stage II on coagulation time of platelets and MPs (*P* >0.05). We found an inverse correlation between clotting time and PS^+^ platelets (*r* = −0.81, *P* = 0.027), MPs (*r* = −0.74, *P* = 0.036), and PMPs levels (*r* = −0.69, *P* = 0.043) in patients. However, there was no correlation between clotting times and EMPs (*r* = −0.84, *P* = 0.062), or TF^+^ MPs levels (*r* = −0.64, *P* = 0.059).

To determine the necessity of exposed PS and TF on platelets and MPs to support coagulation time, we performed the coagulation inhibition assays. Treatment with lactadherin prolonged coagulation time of platelets and MPs to the extent of healthy controls, whereas anti-TF did not significantly affect the coagulation times (Fig. 4b). We further evaluated the ability of platelets and MPs to support fibrin formation using turbidity measurements. Platelets or MPs isolated from colon cancer stage IV resulted in significant fibrin production compared to controls. Lactadherin markedly inhibited fibrin formation, whereas anti-TF antibody did not significantly affect fibrin formation (Fig. 4c and d). These data indicate platelets and MPs trigger PS-dependent fibrin production.

### Confocal microscopy

**Fig. 5:**
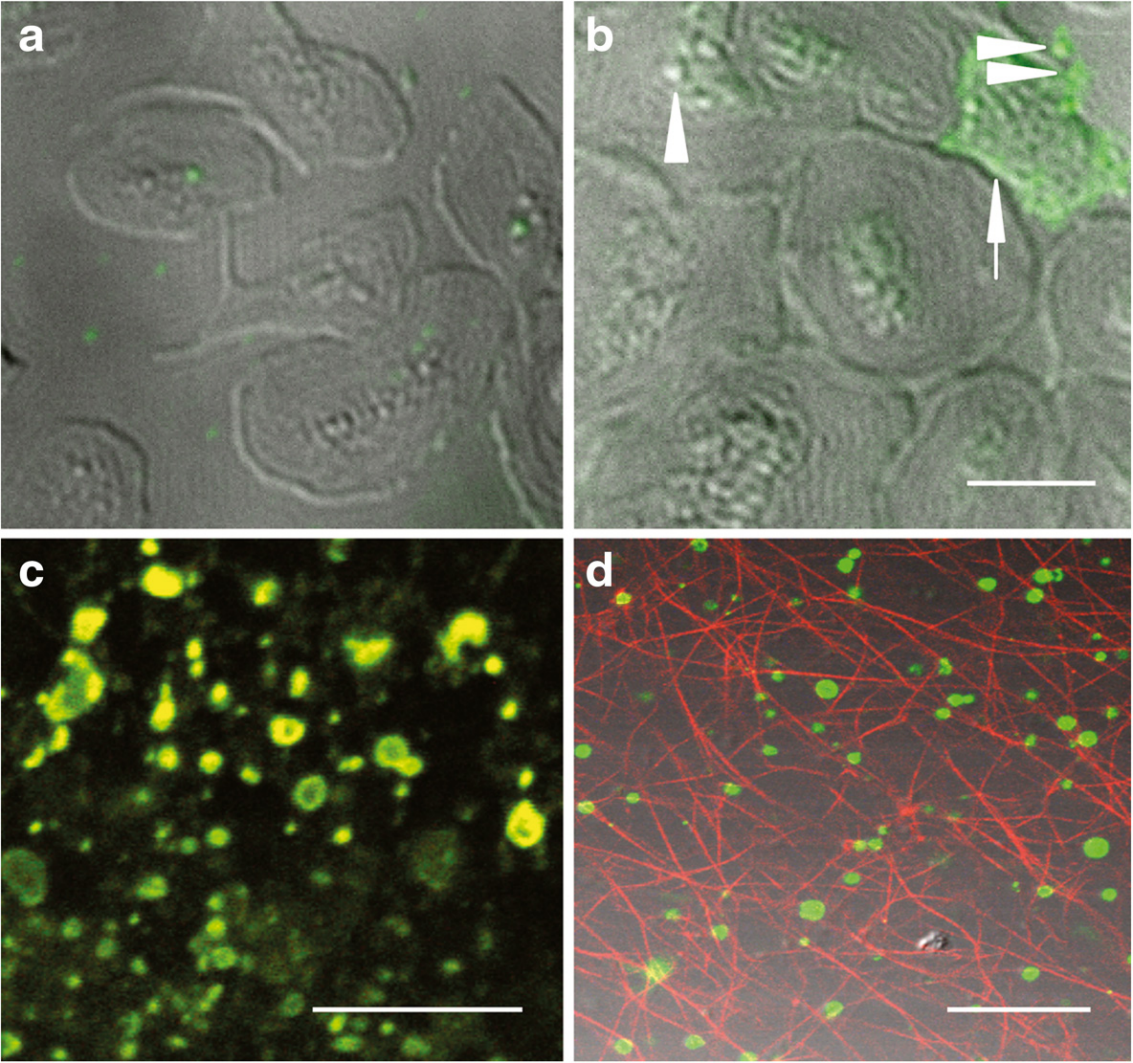
PS exposure on platelets and MPs and fibrin formation was evaluated using fluorescence confocal microscopy. **a** Platelets from healthy subjects barely marked with green fluorescence, **b** while platelets from colon cancer stage IV were labeled with lactadherin (green fluorescence, arrowhead) and PMPs (triangles) were shedding from PS exposed platelets. **c** MPs were strongly labeled with both Alexa 488-lactadherin (green) and Alexa 647-annexin V (red) indicating extensive PS exposure. **d** Fibrin labeled with red fluorescence was observed along with PS^+^ MPs labeled with lactadherin (green). Representative image; scale bars represent 10 μm (**b**) or 5 μm (**c** and **d**)

PS^+^ platelets, MPs and fibrin at colon cancer stage IV were imaged using fluorescence labeled lactadherin (green, Alexa 488), annexin V (red, Alexa 647) and anti fibrin antibody (red, Alexa 647) by laser confocal microscopy. Platelets in healthy individuals were rarely labeled with green fluorescence (Fig. 5a), but platelets from patients stage IV were detected obvious green fluorescence suggesting strong PS exposure (Fig. 5b, arrowhead). Meanwhile, those PS^+^ platelets were releasing small vesiculous and PMPs were shedding from their parent platelets (Fig. 5b, triangles). In colon cancer patients stage IV, large quantities of MPs were generated in the blood samples with extensive exposure of PS as indicated by labeling with both lactadherin (green) and annexin V (red) generating co-localized yellow fluorescence (Fig. 5c). Because cellular PCA dictates fibrin clot formation, we sought to explore whether MPs had a similar effect. MPs isolated from patients stage IV were incubated with normal MDP and fibrin formation was evaluated by red fluorescence anti fibrin antibody. We observed strikingly abundant fibrin spread around MPs, and this resulted in fibrin network formation (Fig. 5d).

## Discussion

Our study demonstrated that plasma levels of circulating PS^+^ platelets and MPs were significantly higher in colon cancer patients with stage I/II/III/IV and stage II/III/IV than in controls, respectively. PMPs and EMPs were significantly increased in stage III/IV and in stage II/III/IV patients, respectively. In addition, we showed that platelets in all stages of colon cancer patients and MPs in stage II/III/IV had higher intrinsic/extrinsic FXa and thrombin production, and shorter coagulation time than ones from healthy subjects. Fibrin formation of platelets and MPs from colon cancer stage IV was higher than that from healthy individuals. The PCA can be successfully blocked by binding of lactadherin to PS on platelets and MPs. These results therefore indicate that PS^+^ platelets and MPs play important role in the hypercoagulable state of colon cancer patients with different stage.

To our knowledge, this is the first study to evaluate the role of PS^+^ platelets in colon cancer patients. PS is normally present on the cytoplasmic leaflet of quiescent cells, with virtually none on the exofacial leaflet [10]. Changing this resting distribution of PS has a strong potential for initiating signaling and was first recognized in the case of platelet activation [9]. Our data showed that PS^+^ platelets were gradually increased from colon cancer stage I to IV. Dymicka et al. showed that patients suffering from colon cancer had high levels of platelet CD62P expression and soluble P-selectin concentration, supporting our findings [14]. Indeed platelet activation and thrombocytosis have been previously reported in those patients and is associated with poor survival [29, 30]. PMPs, as a product of platelet activation, significantly increased in advanced colon cancer patients in our current study. In contrast to our results, another study showed that the amount of CD41a^+^ PMPs did not significantly differ between patients and controls [19]. However, their report was based on a population of less than 20 colon cancer patients and annexin V was used to detect PMPs. Annexin V has been reported to be a less effectively and sensitively probe to detect PS-rich cells and MPs than lactadherin [22, 23]. Moreover, our confocal photomicrographs of MPs showing strong lactadherin staining and rare annexin V staining further strengthen this.

High-grade disease is associated with a considerably increase of nodal and distant metastasis [31]. Patients with stage III/IV were present with enhanced metastasis rate and also have markedly higher levels of PS^+^ platelets and PMPs compared with patients with stage I/II. The exact mechanisms inducing increased levels of PS^+^ platelets and the formation of PMPs in colon cancer are not well understood, but it seems that there is a relationship with platelet-tumor cell cross-talk. The colon cancer cells line can bind to activated platelets, stimulate powerful platelet aggregation, and promote platelet secretion [32, 33]. This may partly lead PMPs shedding from the PS^+^ or activated platelets. PMPs in this study were shedding from PS^+^ platelets may further explain this phenomenon. High plasma level of PS^+^ platelets in colon cancer patients may have the capacity to contribute to different stages of cancer progression and metastasis since activated platelets play an important role in neoplasm angiogenesis, invasion, survival in circulation and metastasis [15, 34–36]. Intriguingly, our results showed that the plasma level of PMPs did not exhibit a tendency analogous to the level of PS^+^ platelets but that levels of PS^+^ platelets and PMPs were both correlated with colon cancer metastasis. Although the angiogenic potential of PMPs is as important as the angiogenic potential of platelets [37, 38], the function of high PMPs levels in advanced colon cancer are still needed to be explored and verified in large population based studies in the future.

High levels of EMPs have been reported in cardiovascular diseases, autoimmune diseases, but not in colon cancer [39, 40]. Our results showed that EMPs increased in colon cancer patient stage II/III/IV but not in stage I. This may due to enhanced tumor spread and angiogenesis in higher-grade colon cancer compared with stage I. The major mediators of tumor angiogenesis are the vascular endothelial growth factor (VEGF) family and its receptors, which have been reported to be closely involved in colon cancer stage II/III/IV [41–43]. VEGF stimulation activates endothelial proliferation, and promotes tumor cell-associated, vessel-mediated and immuno-inflammatory processes in colon carcinoma during which enhanced EMPs would be released [42]. Interestingly, we found that the levels of EMPs were not correlated to coagulation time in colon cancer patients, suggesting that increased levels of EMPs in the patients may have their unique role in those patients. Several studies have shown the involvement of EMPs at different stages of angiogenesis, including matricial degradation, recruitment and differentiation of endothelial progenitors, and proliferation and migration of endothelial cells [44]. Thus, high levels of EMPs may be involved in colon cancer angiogenesis.

TF has been commonly regarded as one of the important procoagulant substance, which was correlated with the risk of thrombosis in cancer patients such as pancreatic cancer [20]. Here, we reported that colon cancer patients have an increased plasma level of TF^+^ MPs. Previous study showed that high levels of TF^+^ MPs in advanced colon cancer patients, supporting our results [19]. In the present study, TF^+^ MPs were also over-expressed in colon cancer stage I. This augment may at least in part due to colon cancer-derived TF^+^ MPs, since tumor-derived MPs are critical sources of TF in cancer and human TF antigen has been demonstrated to be released into the blood from human colon tumor [17, 45, 46]. Increased MP TF activity has been detected in colon cancer patients [20]. However, we found that anti-TF antibody did not significantly prolong the clotting time of MPs, and further coagulation and fibrin inhibition assays suggest that PS^+^ platelets and MPs seems to be the main source of excessive PCA in colon cancer patients. One possible explanation is that plasma-exposed TF is frequently encrypted with little or no detectable PCA and is decrypted through the availability of clusters of PS [21]. Moreover, our finding is also supported by our previous studies showing that the expression of activated TF overlaps with PS exposure and TF-dependent FXa generation was decreased after blocking PS [13, 47].

Hypercoagulability is well documented in all types of cancers, and is the second leading cause of death in cancer patients [48]. Here we demonstrate a hypercoagulable state that is associated with increasing levels of PS^+^ platelets and MPs in those patients. PS exposure on platelets and MPs provides binding sites for FXa, supports thrombin generation, results in fibrin formation, and processes blood clotting [9, 10]. Our data demonstrated a statistically significant difference between colon cancer patients and healthy subjects regarding the procoagulant enzyme complexes, fibrin formation and coagulation time of platelets and MPs. We also observed an inverse correlation between PS^+^ platelets, MPs and PMP levels and the clotting time measured by the functional coagulation time test. PS blockage by lactadherin lengthens the coagulation time and reduced fibrin formation to control levels, and inhibited their intrinsin/extrinsic FXa and thrombin production about 80 %. Taken together, these results strongly reflect that functional PCA of circulating platelets and MPs that is linked to the PS present at the platelet or MP surface.

In colon cancer patients, PCA of platelets and MPs has the similar trend to the level of PS^+^ platelets and MPs separately, which is inconsistent with our previous reports that high PS^+^ platelets had relative high PCA [12, 13]. In our current study, PCA of MPs was slightly higher in patients with stage II colon cancer patients, and obviously augmented in stage III/IV or advanced stage colon cancer. However, Van Doormaal et al. reported that MP phospholipid-dependent PCA was not different between cancer patients and healthy controls [49], but they did not account for the effect of disease stage. The PCA of MPs in stage I patients and healthy controls was similar to those in our study. The PCA of MPs has been reported to be higher in patients with advanced stage breast cancer than in healthy subjects [50], supporting our results. In addition, in cutaneous malignant melanoma, the PCA of MPs not only increased in stage III/IV, but also in stage II [51]. Thus, PCA of MPs makes contribution to hypercoagulable state in cancer patients depending on tumor classification and tumor types. Nevertheless, we can’t rule out other variables such as tumor site or size and these factors need to be further evaluated in colon cancer patients.

There are currently controversies about the antiplatelet treatment of colon cancer patients. Although low-dose aspirin usage after diagnosis of colon cancer did not increase survival time [52], but evidence from randomized controlled trials and meta-analysis of aspirin, to prevent vascular events, indicates that post diagnosis aspirin therapy may improve colon cancer overall survival, and reduce both all cause and colon cancer specific mortality [53–56]. Furthermore, proximal colon cancer patients have been reported to be greatest benefit for long-term effect of aspirin. Our data suggests that high level of PS exposure on platelets and MP is associated with increased PCA in different stage of colon cancer patients. It seems crucial to block PS, as well as FXa and thrombin, to prevent the activated blood coagulation of platelets and MPs. Antiplatelet treatment is not sufficient to entirely prevent PCA activity in stage II/III/IV since there is a significantly high level of MPs and MP-associated PCA especially in advanced colon cancer. Thus, the administration of both anticoagulant and antiplatelet therapy seems to be necessary. The results from this study suggest that future research should focus on discovery and development of direct PS inhibitors.

In our study, we only investigated PS^+^ platelets, MPs, PMPs, and EMP and we cannot exclude the increased PS exposure on other cells or the presence of MPs from other cell origin, that is, inflammatory, immune, or tumor cells. Furthermore, considering the small population of study subjects, we are unable to conclude that PS^+^ platelets or MPs or their PCA can be used a biomarker of risk in these patients. Prospective large cohort studies are underway to confirm the exact role or value of PS^+^ cells or MPs in colon cancer patients. In conclusion, this study demonstrates high levels of circulating PS^+^ platelets, MPs, PMPs, EMPs and TF^+^ MPs in colon cancer patients in association with a significant increased PCA. Further studies are required to assess both the prognosis and the thrombogenic value of PS^+^ cells and MP levels in these patients and to determine whether these cells or MPs can also originate from other cell types.

## Conclusion

Collectively, our results show that PS platelets and microparticles promote PCA in colon cancer patients and may unveil a potential therapeutic target to prevent coagulation in those patients.

## Abbreviations

EMPs: Endothelial-derived MPs
HS: Healthy subjects
MDP: MP-depleted plasma
MPs: Microparticles
PCA: Procoagulant activity
PFP: Platelet-free plasma
PMPs: Platelet-derived MPs
PRP: Platelet-rich plasma
PS: Phosphatidylserine
TF: Tissue factor
